# Combined paraganglioma and IgG4-related retroperitoneal fibrosis

**DOI:** 10.1016/j.radcr.2024.04.065

**Published:** 2024-05-17

**Authors:** Maryam Bolouri, Perry Veras, Shashank Gupta, Yuliya Zayats, Emad Allam

**Affiliations:** Loyola University Medical Center and Loyola University Chicago, 2160 S First Ave, Maywood, IL 60153, USA

**Keywords:** Paraganglioma, Neuroendocrine tumor, Retroperitoneal fibrosis, IgG4-related disease

## Abstract

A paraganglioma is a neuroendocrine tumor that may secrete catecholamines and present with symptoms of sympathetic overload such as hypertension and diaphoresis. It is important that paragangliomas are identified, as they must often be treated by surgical excision. IgG4-related retroperitoneal fibrosis (IgG4-RPF) is a systemic inflammatory disease that results in the infiltration of IgG4-positive plasma cells in the retroperitoneum. Such fibrosis may adversely affect nearby organs and tissues. Here, we describe a case of combined paraganglioma and IgG4-RPF in a 47-year-old female patient. This case demonstrates the deleterious effect of these two conditions when they occur simultaneously.

## Background

Paragangliomas are rare neuroendocrine tumors arising from extra-adrenal autonomic paraganglia [1]. These tumors are made of chromaffin cells and may secrete catecholamines [2]. Functional paragangliomas are hormone-secreting, while non-functional tumors do not secrete hormones [3]. Catecholamine-secreting paragangliomas present similarly to pheochromocytoma with symptoms such as hypertension, headache, diaphoresis, and tachycardia [1]. Treatment typically involves surgical resection of the tumor [4].

IgG4-related retroperitoneal fibrosis (IgG4-RPF) is a fibroinflammatory immune-related disease characterized by mass-forming inflammation, fibrosis, and IgG4-producing plasma cells in the retroperitoneal space [5]. This inflammation may damage or disrupt surrounding blood vessels, nerves, and ureters [5]. Patients present with nonspecific symptoms including back pain, abdominal pain, and lower extremity edema [5].

Here, we describe a case of combined retroperitoneal paraganglioma and IgG4-RPF in a 47-year-old female patient. IgG4-RPF may have a compounding effect on the symptoms of paraganglioma and impact nearby structures, complicating surgical resection of the tumor.

## Case presentation

A 47-year-old female with a past medical history of hypertension and gastroesophageal reflux disease (GERD) presented to the emergency room with 4-5 months of abdominal pain. The pain was described as a burning sensation in the periumbilical area with radiation to the back bilaterally. The pain would sometimes wake the patient up while sleeping. The patient had a history of cholecystectomy and cesarean section. She had no genetic syndromes or relevant family history. She denied sweating, palpitations, and headaches. Her hypertension had been difficult to control with medications.

**Fig. 1:**
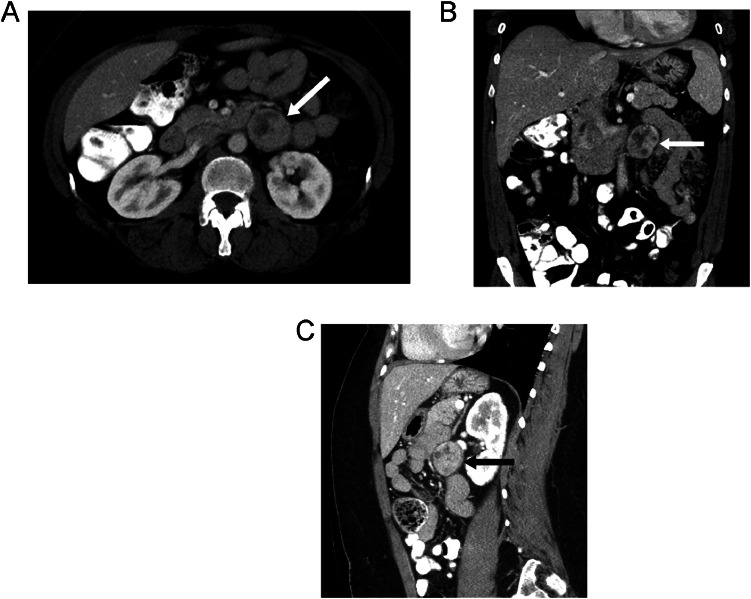
(A) Axial, (B) coronal, and (C) sagittal CT images of the abdomen with IV contrast demonstrate a round, well-circumscribed, heterogeneously enhancing lesion (arrows) located in the left retroperitoneal space adjacent to the aorta and abutting the caudal margin of the left renal vein. This did not appear to arise from any of the adjacent visceral or vascular structures.

**Fig. 2:**
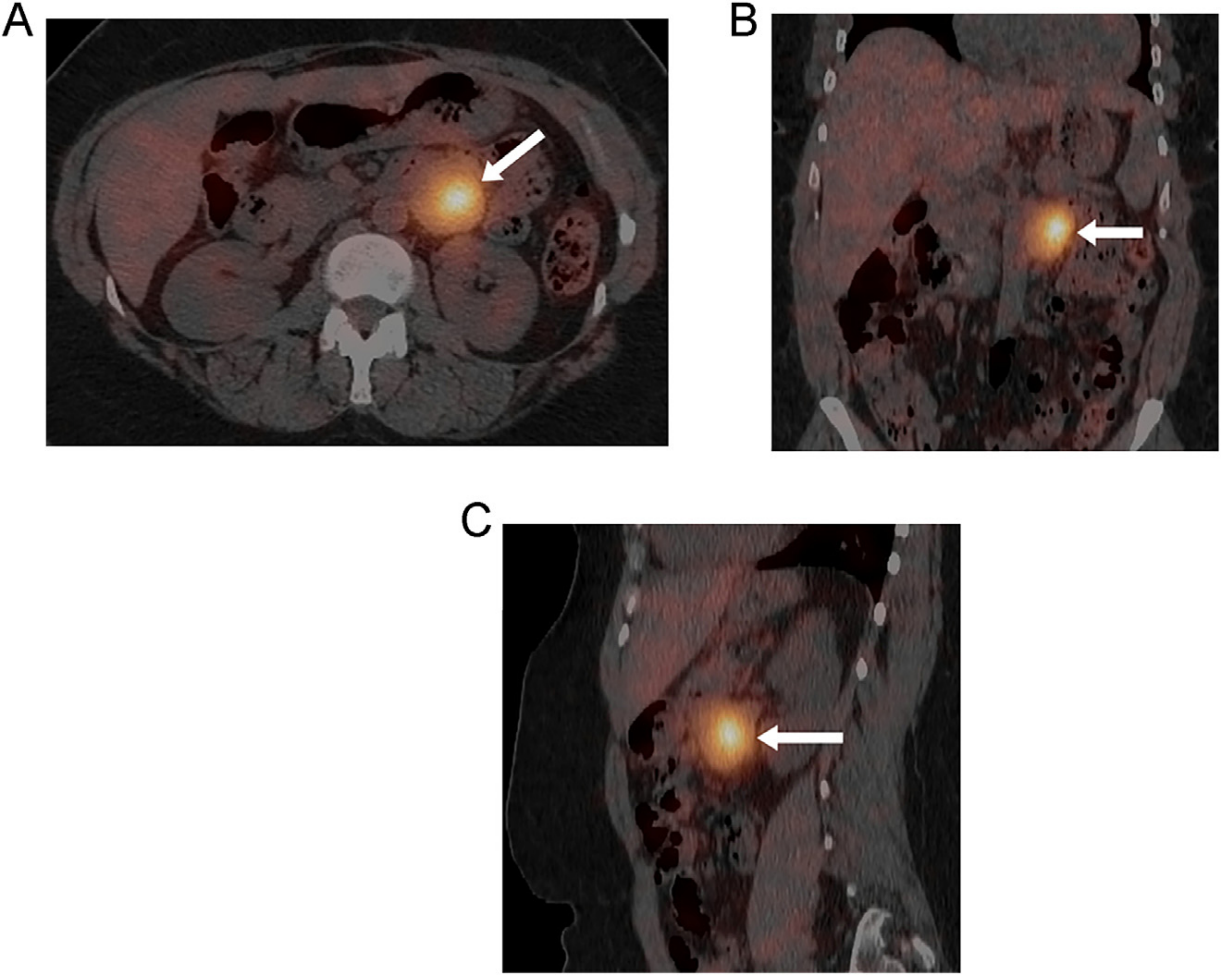
(A) Axial, (B) coronal, and (C) sagittal SPECT/CT images of the abdomen from an I-123 MIBG scan demonstrate radiotracer uptake in the left retroperitoneal lesion (arrows), confirming a neuroendocrine tumor. The uptake is most pronounced in the central to left aspect of the lesion, corresponding to the enhancing portion seen on the prior CT.

CT was performed to evaluate for a cause of abdominal pain while the patient was in the emergency department. The CT revealed a 3.2 cm round circumscribed mass in the left retroperitoneal space adjacent to the aorta and abutting the caudal margin of the left renal vein (Figs. 1 A-C). Biochemical testing revealed elevated plasma metanephrines and chromogranin A, suggestive of a neuroendocrine tumor. I-123 metaiodobenzylguanidine (MIBG) nuclear medicine test was performed to assess for a neuroendocrine tumor. The MIBG test was positive and confirmed a functioning neuroendocrine tumor in the retroperitoneum anterior to the mid-pole of the left kidney (Figs. 2 A-C). Note that there was heterogenous enhancement and heterogenous radiotracer uptake in the mass. No other mass or lymphadenopathy was detected.

The patient underwent a pre-operative course of alpha blockade and subsequently underwent surgical resection of the mass. A left ureteral stent was placed. Pathology was consistent with a paraganglioma as well as extratumoral plasma cell infiltrate, fibrosis, and phlebitis, highly suggestive of IgG4-RPF, which was confirmed by immunostaining and in-situ hybridization. The margins of the surgical resection were free of tumor.

**Fig. 3:**
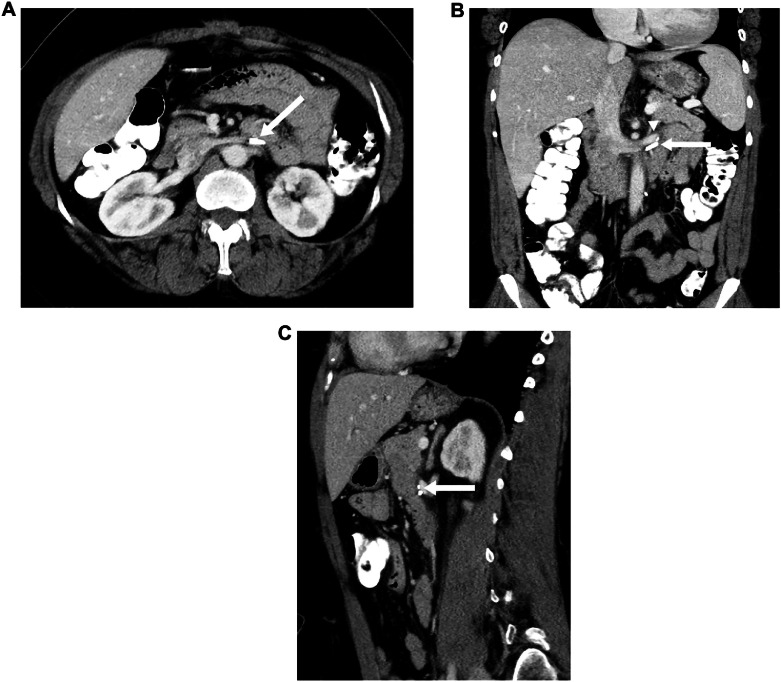
(A) Axial, (B) coronal, and (C) sagittal CT images of the abdomen with IV contrast 1 year after surgery demonstrate resection of the retroperitoneal mass. There is no evidence of residual or recurrent tumor. Surgical clips (arrows) are seen at the resection site, including two surgical clips along the left renal vein (arrowhead).

The post-operative course was complicated by uncontrolled pain requiring epidural placement, urinary retention requiring Foley catheter placement, and narcotic-induced ileus requiring nasogastric decompression. These were all resolved at the time of discharge from the hospital, and at 6-week follow-up the patient reported improvement in abdominal pain. The patient is doing well without symptoms or tumor recurrence 4 years after the surgery (Fig. 3).

## Discussion

A paraganglioma is a rare neuroendocrine tumor. While pheochromocytomas arise from the adrenal medulla, paragangliomas originate outside the adrenal gland and are commonly seen in thoracic and abdominal sympathetic nerves [4]. The combined incidence of pheochromocytoma and paraganglioma is less than 1 in 300,000 [1]. The typical presentation of paraganglioma is a triad of sweating, palpitations, and headache, although it can have a variety of presentations and is therefore referred to as the great masquerader. Paraganglioma is commonly identified by CT, and functional imaging such as I-123 MIBG can be used to confirm the diagnosis. These lesions are highly vascular and prone to hemorrhage and necrosis. Therefore, on CT, paraganglioma may appear as a heterogeneously enhancing lesion [2]. Medical blockade through use of alpha-receptor blockers must be used prior to surgery to avoid intra-operative hypertensive crisis [2].

IgG4-related disease encompasses a variety of conditions affecting a range of organ systems. In this patient, we note the presence of IgG4-RPF, one of the most common manifestations of IgG4-related disease [5]. IgG4-RPF typically presents with poorly localized pain in the lower back and abdomen with nocturnal exacerbation, as seen with our patient [5]. It manifests as fibroinflammatory masses in the retroperitoneal space, typically along the abdominal aorta, iliac arteries, and the ureters, as seen in this case with the need for left ureteral stent placement [5]. Corticosteroids are the first-line treatment for IgG4-RPF.

While autoimmune disease tends to be more common in females, IgG4-RPF has a male: female ratio of 3:1 [5]. IgG4-RPF was not the initial suspicion for this case, but it likely compounded her symptoms, particularly her abdominal pain. The combined occurrence of paraganglioma and IgG4-RPF in this case indicates the need for a multifaceted approach to diagnosis and treatment. Sympathetic ganglia are present in the retroperitoneal space, and therefore it is a potential location for functional paragangliomas, in addition to being the pathognomonic site for IgG4-RPF. Although rare, there are some case reports which link carcinoid tumors with RPF, potentially due to the desmoplastic reaction stimulated by the tumor [6]. A case of pheochromocytoma within IgG4-related disease has also been reported in the literature [7]. It occurred in a 36-year-old female who presented with abdominal pain. Imaging studies including CT, MRI, and ^18^F-FDG PET-CT showed a large 9 cm heterogenous right adrenal mass with a fibrotic capsule. Urinary metanephrines and normetanephrines were elevated, and serum IgG4 was elevated. The mass was resected and pathologic criteria confirmed pheochromocytoma wrapped in a fibrous capsule containing IgG4-positive cells.

This is the first reported case of extra-visceral neuroendocrine tumor with associated IgG4-RPF. It is important to look for signs of RPF when a neuroendocrine tumor is identified and vice versa. Laboratory testing may be warranted if the lesion appears extra-visceral, atypical/markedly heterogenous, or there is evidence of surrounding fibrosis on multimodal imaging.

## Conclusion

While extremely rare, paraganglioma and IgG4-RPF may occur concurrently, complicating symptoms, diagnosis, and treatment. Our case indicates the importance of identifying retroperitoneal fibrosis, as it necessitated stent placement during surgical resection of the paraganglioma in this patient. To our knowledge, there have been no other reported cases of composite paraganglioma and RPF within the same location in the retroperitoneum.

## Patient consent

Informed consent for this case was obtained from the patient.
